# Resilience, Risk and Protective Factors in Children and Adolescents Growing Up with Parental Alcohol Misuse

**DOI:** 10.1007/s40653-025-00776-0

**Published:** 2025-10-27

**Authors:** Kristel Svendal, Oyeniyi Samuel Olaniyan, Gaby Ortiz Barreda

**Affiliations:** 1 Department of Health Promotion and Development, University of Bergen, Bergen, Norway; 2 Department of Psychology, Inland Norway University of Applied Sciences, Lillehammer, Norway; 3 Master in Child Protection, University of Bergen, Bergen, Norway

**Keywords:** Parental alcohol misuse, Resilience, Scoping review, Protective factors, Risk factors

## Abstract

The present scoping review addresses resilience, risk/protective factors, and coping in children growing up with parental alcohol misuse. Around 8.7 million children in the USA live with a parent who misuses alcohol, and between 780,000 and 1.3 million children in the UK live in families affected by alcohol misuse. Our preliminary search indicated that no existing global statistics are available on children living with parental alcohol misuse, which underscores the importance of expanding knowledge in this field. On the one hand, research shows that growing up in homes where one or two parents have an alcohol use disorder can lead to a variety of challenging outcomes in adulthood for the children. On the other hand, there are children who face these hurdles in their childhood but manage their adulthood better than expected. Twenty-five primary studies were included. These studies were conducted in different parts of the world. Findings are discussed in view of attachment theory and regulation theory as a framework for understanding resilience, risk/protective factors, and coping in children and adolescents growing up with parental alcohol misuse. We identified risk and protective factors on biological, individual, parental, familial, and social levels. Each level addresses factors that affect children’s physical and psychological development, both positively and negatively. Furthermore, the analysis included research on interventions implemented to prevent negative consequences for the children involved. Analysis of the literature indicates that further research on familial and social levels is required.

**Supplementary Information:**

The online version contains supplementary material available at 10.1007/s40653-025-00776-0.

## Introduction

Alcohol misuse constitutes a significant global health problem, with severe health consequences for the individual using alcohol, as well as anxiety, trepidation, and uncertainty amongst their immediate family and society. In 2020, approximately 1.34 billion individuals worldwide consumed harmful amounts of alcohol (Bryazka, Dana et al., 2020). Alcohol misuse is ranked among the top five risk factors leading to mortality globally (WHO, 2024). In 2016, deaths related to excessive alcohol consumption were estimated at approximately 3.3 million worldwide, representing about 5.9% of the total global deaths (WHO, 2018).

The World Health Organization (WHO) defines alcohol misuse *“…as a pattern of alcohol use that is causing damage to health*,* and the damage may be physical (as in cases of liver cirrhosis) or mental (as in cases of depressive episodes secondary to heavy consumption of alcohol)”* (WHO, 2014). Alcohol misuse is considered the most harmful drug in terms of the damage inflicted on individuals surrounding the person with the disorder despite the fact that it does not differ significantly from other forms of substance misuse (Nutt et al., 2010). Europe has consistently recorded the highest level of alcohol consumption compared to other regions (WHO, 2018; Bryazka, Dana et al., 2020). Economic factors may significantly contribute to this trend, as societies and individuals with higher economic status have greater access to alcohol (WHO, 2018; Probst et al., 2020). Studies suggest that alcohol misuse varies significantly by age and location (Bryazka, Dana et al., 2020; Probst et al., 2020). The study by Bryazka, Dana et al. (2020) states that stronger interventions towards younger people are necessary to reduce the global health loss attributable to alcohol misuse.

Despite the absence of global statistics on children living with parental alcohol misuse, some regional data highlight the significant impact of this problem. The National Surveys on Drug Use conducted between 2009 and 2014 indicate that approximately 8.7 million U.S. children under the age of 17 (roughly 10.5% of children in this age group) reside with at least one parent who misuses alcohol (Lipari & Van Horn, 2017). Additionally, it is estimated that between 780,000 and 1.3 million children in the United Kingdom live with parents who have an alcohol problem. Given the vulnerability of many children growing up in adverse living conditions, it is crucial to acknowledge that numerous cases remain unregistered (Prime Minister`s. Strategy Unit, 2004).

## Background

Alcohol misuse is primarily determined by social conventions and varies across cultures. However, irrespective of social conventions, children have rights that must be considered, and these rights are enshrined in the UN’s Convention on the Rights of the Child (UN General Assembly, 1989). Among other things, the child has a fundamental right to protection, participation and provision. Negligence and violations of children’s rights can result in a diminished quality of life and reduced opportunities for development and participation in society (Linden, 2004). While the childcare exhibits significant cultural variations, there is an evolutionary biology and systems theory basis that identifies a common core of care functions for children. These functions include feeding, regulation, protection, communication, and stimulation (Brandtzæg et al., 2014).

Growing up with alcohol-misusing parents can deprive children of essential care functions, and this may have a devastating effect on their growth and development. One of the common characteristics of parents who misuse alcohol is their lower involvement in interactions with their children, which are often characterized by low energy and lack of reciprocity. Consequently, children may perceive their parents as unreliable and unpredictable (Kvello, 2010).

### Resilience and Coping in Children Raised in Families with Alcohol Misuse

Despite adverse conditions at home, many children and young people demonstrate resilience (the ability to cope and thrive in the face of adversities). Resilience can be further defined as a positive adaptation despite experiences of adversity or trauma (Garrido-Hernansaiz et al., 2020).

According to Rutter (2016) resilience refers to *“the finding that some individuals have a relatively good psychological outcome despite suffering risk experiences that would be expected to bring about serious sequelae”.* Traits or processes may be described as resilient only if they lead to a positive adaptation after a serious adversity. Consequently, only individuals who have experienced serious adversity can be considered resilient (Glennie, 2010). In recent years, resilience has garnered increased attention, and efforts have been geared towards identifying, describing, and understanding this phenomenon. Furthermore, evidence regarding the role of resilience in coping and adaptation has prompted a critical appraisal of preventive and therapeutic measures designed to increase resilience (Borge, 2007).

Coping and resilience are interrelated concepts, and both focus on responses to stress. Coping strategies play a crucial role in determining the degree of resilience (Garrido-Hernansaiz et al., 2020). However, these concepts are distinct. Coping involves a set of skills, whereas resilience denotes a successful outcome resulting from exercising those skills (Compas et al., 2001).

### Risk and Protective Factors in Children Raised in Families with Alcohol Misuse

Previous research suggests that biological, individual, and social factors play a crucial role in the development of resilience (Borge, 2007), and the interplay between risk and protective factors at individual and environmental levels is integrated into its definition. Although the concept of resilience is associated with risk factors, exposure to these factors does not predict a negative outcome with absolute certainty (Tusaie, 2004). Children who grow up in stressful environments, where one or two parents have an alcohol use disorder, may be at increased risk of experiencing multiple negative events throughout their lifetime (Lacopetti et al., 2018).

Research has shown that children of alcohol-misusing parents are at higher risk for behavioural, cognitive and physical problems (Simonic & Osewska, 2023; Liberman, 2000; Johnson & Leff, 1999). Genetic influences have been identified as the largest contributor to the variance in alcoholism risk (Heath et al., 1997; Anda et al., 2002; Sørensen et al., 2011). Children of alcohol-misusing parents are also at an increased risk of adverse outcomes, including a higher likelihood of emotional and behavioural maladjustments. These may include aggression (Eiden, 2018), lower school attendance, and poorer academic self-concept (Lowthian, 2022; Andreas et al., 2022). Having an alcohol-misusing parent can significantly strain children, leading to lasting cognitive and emotional problems that may persist into adulthood (The National Institute of Public Health, 2018; Simonic & Osewska, 2023).

Research has identified the importance of emotional support, open communication and positive values ​​at the family level as protective factors that contribute to family resilience (Coyle et al., 2009). Wlodarczyk et al. (2017) offer an extensive overview of protective mental health factors in children of parents with alcohol and drug use disorders. Their systematic review highlights the relevance of social support as a crucial protective factor for children’s mental health. Due to the lack of research on protective mental health factors in children of alcohol-misusing parents, Wlodarczyk et al.(2017) call for more longitudinal studies that include both risk and protective factors for mental health.

Research on resilience, risk and protective factors in these children is vital for informing future interventions aiming to mitigate their risk factors. The present study aims to enhance the understanding of resilience and the factors affecting children growing up in households with alcohol-misusing parents by summarizing literature on alcohol use disorder as well as identifying resilience and risk/protective factors.

### Present Study

By answering the questions below, the present paper seeks to expand our current knowledge base on children growing up in households with alcohol misuse.

1. What does empirical research teach us about resilience in children and adolescents growing up in families with alcohol misuse?
2. What are the common risks and protective factors in children and adolescents who grow up in these homes?

Scoping reviews are designed to *“follow a systematic approach to map evidence on a topic and identify main concepts*,* theories*,* sources*,* and knowledge gaps”* (Tricco et al., 2018). Research has shown the importance of scoping reviews in knowledge synthesis, particularly in areas where an overview of the literature is difficult (Colquhoun et al., 2014; Daudt et al., 2013; Grant, 2009; Levac et al., 2010; Pham et al., 2014). The present study will therefore follow the methodology for conducting a scoping review as proposed by Arksey and O’Malley (2005): (1) identify the research question, (2) identify relevant studies, (3) study selection, (4) charting the data, (5) collating, summarizing and reporting the results.

## Method

### Stage 1: Identify the Research Question

The present scoping review aims to explore what empirical research exists about resilience, risk and protective factors in children and adolescents growing up in families with alcohol misuse. Our first search was done in 2018 as part of the first author`s master thesis. We begin with wide search criteria to establish what type of evidence was available before narrowing the search. A follow-up search was conducted again between February and April 2023, with the purpose of updating and identifying new scientific literature produced during that period.

**Table:** **Box. 1** Keywords used in literature search

Alcohol misuse	Children	Protective factors and resilience
“Alcoholic parents” OR “Alcoholic father” OR “Parental alcoholism” OR “Parent’s Alcoholism” OR “Parental alcohol use disorder” OR “Parental alcohol use” OR “Father’s alcoholism” OR “Sons of male alcoholics” OR “Parental alcohol problems” OR “Children of alcoholics” OR “Alcoholic families” OR “Familial alcoholism” OR “Parenting in alcoholics” OR “Parents’ Drinking Problems” OR “Drinking parent” OR “Parental drinking problem” OR “Parents with drinking problems” OR “Parent alcohol-use” OR “Children of heavy-drinking parents” OR “Mothers with a Drinking Problem” OR “Parents with alcohol abuse” OR “Alcohol-dependent parents” OR “Parental abusive drinking” OR “Parental problem drinking” OR “Parent drinking” OR “Alcoholic” fathers” OR “Parents with alcohol abuse”	Child OR Children OR Adolescent OR Young OR “Young adult” OR Kids OR Youngster OR Minor	Resilience OR Resilient OR Coping OR Adjustment OR “Coping strategies” OR “Coping skills” OR “Protective factors” OR “Emotional regulation strategies” OR “Regulation-abilities” OR “Emotional resilience” OR “Behavioral Resilience” OR “Children’s coping strategies”

### Stage 2: Identify Relevant Studies

This stage involves identifying the relevant studies and developing a plan and search strategy. Some essential characteristics of the articles, such as year of publication, language and terms, were collected at this stage (Arksey & O’Malley, 2005). Subsequently, we conducted searches on the following databases: Web of Science, PsycINFO and MEDLINE. These databases were chosen because they are known as large databases containing medical, psychological and social science literature and articles. We identified three main thematic filters with several different keywords in each filter. The search included studies that were published between 2005 and 2023.

### Stage 3: Study Selection

The first author screened article’s titles, keywords, and abstracts in light of the search terms that were developed. A total of 383 were retrieved from the three databases, PsycINFO, MEDLINE, and Web of Science. Only 43 full text articles were assessed and 25 of these were included in the final analysis. In our first search in 2018 yielded 330 documents from the three databases: 106 from Web of Science, 140 from MEDLINE and 84 from PsycINFO. Of these, 51 were not research articles or were articles in a language other than English. These were therefore excluded. Additionally, 26 duplicates were also excluded. In the initial exploration of relevance, 161 articles were deemed irrelevant based on title and abstract and were excluded. The 92 remaining articles were assessed based on the study’s objectives and inclusion criteria and a further 59 articles were excluded. A total of 33 articles were read in full, 14 did not meet the criteria to be included in the study. Of these 14 articles, some focused to a greater extent than desired on other mental disorders in parents, or on drug use other than alcohol. Two of the studies were ongoing intervention studies that had no clear findings.

**Fig. 1:**
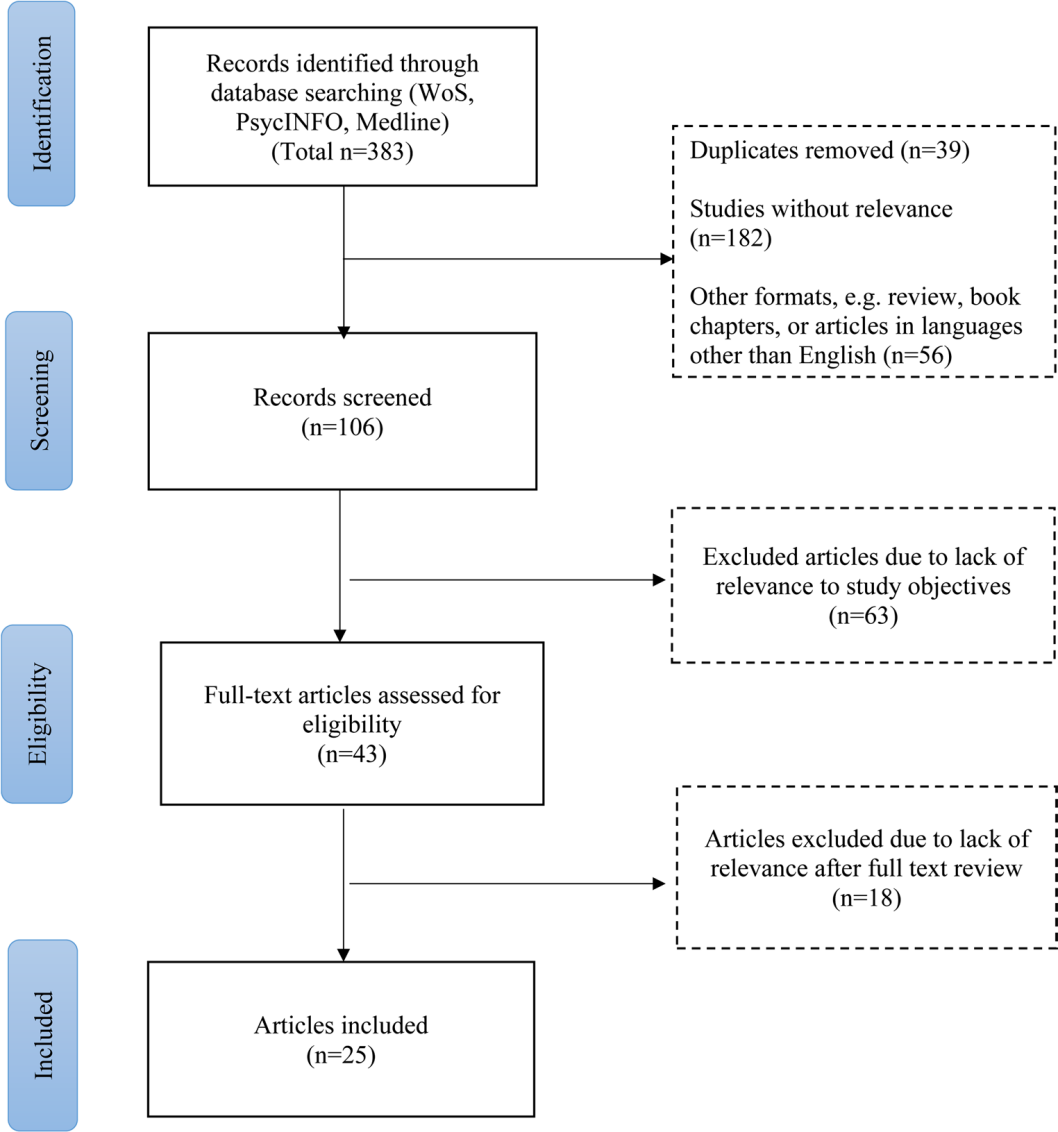
Flow diagram of literature identification path

In our follow-up search in 2023, 53 documents were retrieved from the three databases. Thirty were from the Web of science, 2 from MEDLINE and 21 from PsycINFO. Of these, 13 were duplicates and were subsequently excluded. Five were in formats other than academic articles and were therefore also excluded. In the initial exploration of relevance, 21 articles were excluded based on title and abstract. The remaining 14 were assessed further based on the study’s objectives and inclusion criteria. Four of these were excluded due to focus on other drugs than alcohol or failure to meet inclusion criteria. Ten full text articles were read, 4 of these did not meet the inclusions criteria and were therefore excluded. Therefore, 19 articles were included from the 2018 search, and 6 articles were included from the 2023 search, providing a total of 25 articles. See Figure 1.

**Table:** **Box. 2** Inclusion and exclusion criteria

	Included	Excluded
**Databases**	Web of Science, MEDLINE, PsycINFO	Other databases
**Time period**	2005–2023	Articles published before 2005
**Publication type**	Published articles	Books, master’s theses, other formats such as reviews, comments to the editor, duplicates and articles not related to the topic
**Focus**	Studies that focus on resilience and risk/protective factors in children or adult children of parents with alcohol use disorder, parent-child relationship and influencing factors for resilience.	Studies that focus on drugs other than alcohol, vulnerable groups other than children of alcohol-misusing parents and studies not relevant to resilience/risk and protective factors.
**Language**	English	Other languages

### Stage 4: Chart the Data

We developed a data charting form before collecting the data from the included studies in the analysis. Subsequently, information about author, year, country, study aim, research design, sample size, and key findings were collected. The first author compiled all the results into tables with help from co-authors.

### Stage 5: Collate, Summarize and Report Results

The last stage recommended by Arksey & O’Malley (2005) is to collate, summarise and report the results. We organised the results into themes relevant to the research questions and the focus of the study.

## Results

### Study Characteristics

Of the 25 included studies, 16 were published in the USA (Drapkin et al., 2015; Einsberg et al., 2010; Grace-Cleveland et al., 2008; Hall, 2008; Haverfield & Thiess 2020; Haverfield & Thiess 2017; Haverfield & Thiess, 2016; Haverfield & Theiss 2014; Heitzeg et al., 2008; Klostermann et al.,^2011^; Lee & Cranford, 2008; Lee & Williams, 2013; Moe & Wade 2007; Park &. Schepp 2017; Smith et al., 2006; Wong et al., 2018). The remaining 9 were published in the following countries: Malta (Bugeja & Galea, 2022), Canada (Coyle et al., 2009), India (Hebbani et al., 2020), South Korea (Hyun et al., 2010; Namkoong & Cheon 2008 Park & Schepp, 2018), South Africa (Breda & Mushonga, 2021), Russia (Spivakovaskava & Lutsenko 2021), and Lithuania (Tamutiene & Jogaite, 2019). 

### Synthesis of Results

The articles analysed include both quantitative and qualitative studies. Most qualitative studies focused on protective factors concerning the development of children with alcohol-misusing parents. Findings were arranged into six topics: genetic and cognitive differences, children’s innate characteristics, coping, the impact of lack of attachment and communication in alcohol-misusing families, alcohol misuse as an influence on family dynamics, and the importance of social support. The risk and protective factors are addressed within each section. Findings also included studies on interventions to improve the lives of these children. See Appendix Table 1.

### Genetic and Cognitive Differences in Children from Alcoholic Families

Three of the included studies investigated themes related to genetic and cognitive differences (Wong et al.,^2018^; Namkoong et al.,^2008^; Heitzeg et al., 2008). These studies found that children of alcohol-misusing parents are more prone to difficulties such as emotional problems and behavioural difficulties compared to other children, but there are individual differences.

Namkoong et al. (2008) examined genes’ role in relation to children of alcohol-misusing parents. Serotonin transporter gene, dopamine D2 (DRD2), dopamine D4 (DRD4), and GABAA receptor b3 subunit gene (GABRB3) were examined. Dopamine D2 is a protein that is found naturally in the body, and which plays a role in how susceptible a person is to substance use problems (Namkoong et al., 2008). The results showed that the prevalence of dopamine D2 (DRD2) was higher in children of alcohol-misusing parents. In addition, dopamine D4 (DRD4) was less present in these children compared to other children. The presence of GABAA receptor b3 subunit gene was higher in children of alcohol-misusing parents compared to other children. These results support that children growing up in the presences of alcoholism were at greater risk of developing behavioural difficulties and impulsive behaviour patterns compared to others. According to Namkoong et al. (2008), higher levels of dopamine D2 (DRD2) were associated with children growing up around alcoholism being more prone to developing alcohol or other substance misuse themselves, as (DRD2) can be seen as regulating for impulsive sensation-seeking.

In line with Namkoong et al. (2008) a study by Heitzeg et al. (2008) showed differences in the brains of children of alcohol-misusing parents compared to children of parents without alcohol problems. The aim of this study was to identify mechanisms in the brain that can provide insights about protective factors and risk factors in children of alcohol-misusing parents. They were categorized as either vulnerable or resilient depending on the extent of problematic alcohol use during adolescence. The findings indicated that the vulnerable group had more behavioural problems than the resilient group and the control group and were also more antisocial. The study highlighted that the vulnerable group had more activation in the prefrontal cortex than the resilient group, as well as less activation of the ventral striatum and amygdala in response to emotional stimuli. Based on this, the results showed that the resilient group responded better to emotional stimuli, indicative of better emotion regulation and increased flexibility in emotional and social behaviour.

Further, Wong et al. (2018) found that sleep problems in children with alcohol-misusing parents can impact the cognitive part of the brain, leading to increased risk of eventually developing alcohol use problems themselves. The authors also found that that a healthy sleep pattern at an early age provides better self-control/self-regulation in adolescence and is indicative of resilience in adulthood. At the same time, the study showed that in general a good sleep pattern has a seemingly positive effect on resilience and emotional regulation in the short term.

### Children’s Innate Characteristics as Important for Resilience

Five of the included studies explored themes focused on children’s innate characteristics, such as personality, IQ, and attention skills (Lee & Cranford, 2008; Einsberg et al., 2010; Park & Shepp, 2018; Moe et al., 2007; Haverfield & Theiss, 2014). These characteristics also included temperament and self-esteem, qualities that are seen as important for endurance and perseverance in stressful situations (Park & Shepp, 2018; Moe et al., 2007; Haverfield & Theiss, 2014).

Lee and Cranford (2008) investigated which personal characteristics contribute to the development of emotional problems and behavioural difficulties in adolescents of alcohol-misusing parents. The connection between the parents’ alcohol problem and behavioural difficulties was significant. According to Lee and Cranford (2008), the findings indicate that although parents’ alcohol use influences the child’s behaviour, this effect can be moderated by the adolescents’ IQ, personality, characteristics, and resilience.

Other studies found that children of alcohol-misusing parents generally had more impulsivity and lower levels of “*effortful control*” compared with the control group (Einsberg et al., 2010). However, having goals and dreams, academic mastery and schooling may all be protective factors, and adequate academic abilities increase feelings of mastery and self-confidence (Park & Schepp, 2018). Two studies (Haverfield & Theiss 2014; Spivakovskaya & Lutsenko 2021) investigated emotional attributes in adult children of alcohol-misusing parents. Findings from these studies indicate that these children employ different strategies like escape, avoidance and acceptance of responsibility, and that they can overcome their negative emotions.

### Coping

Six of the included studies addressed coping strategies in children of alcohol-misusing parents (Smith et al., 2006; Klostermann et al., 2011; Drapkin et al., 2015; Park & Schepp, 2017; Spivakovskaya & Lutsenko 2021; Mushonga & Van Breda, 2021). Smith et al. (2006) found that children who had supportive and disciplinary parents used effective coping strategies to a greater extent. In addition, these children had a lower degree of emotional and behavioural difficulties. Klostermann et al. (2011) also found that students with alcohol-misusing parents had more depressive symptoms, which in turn poses a risk of developing major depression in adulthood. In addition, this group used less effective coping strategies that resulted in higher consumption of smoking, drinking and other drug use.

Drapkin et al. (2015) also investigated coping styles in adult children of alcohol-misusing parents. There were significantly higher alcohol-related problems and more passive coping strategies in these adults compared to other adults. Gender differences were also identified, were women used more positive coping strategies compared to men (Drapkin et al., 2015). These findings support Klostermann et al. (2011)’s study, but in contrast, Drapkin et al. (2015) found no difference in depressive symptoms in the two groups. Spivakovskaya & Lutsenko (2021) also identified coping mechanisms in adult children of alcohol-misusing parents. These where: Keeping a diary, participation in a rehabilitation program, communicating with friends and grandparents, participating in sports and maintaining a healthy lifestyle. Those who kept a diary said that journaling helped them to understand their emotions and sometimes to overcome feelings of guilt and anger.

### The Impact of Lack of Attachment and Communication in Alcoholic Families

Only three of the included studies examined themes involving attachment and communication (Haverfield & Theiss 2014, 2017, 2020). All three, published by the same authors, demonstrated that a negative parent-child relationship poses a greater risk to the child. The authors explored how communication between parents and children affects emotion regulation and how emotion regulation affects resilience in children of alcohol-misusing parents. They argued that a child’s way of regulating emotions is learned through communication with parents or other caregivers (Haverfield & Theiss, 2017). Findings indicated that parents’ attentive response associated positively with the child’s emotion regulation, and that parents with an alcohol use disorder demonstrated poorer response and regulation for their children, compared to other parents. When the children of alcohol-misusing parents were first regulated by their parents, these children appreciated the parental response even more compared to the children of parents without alcohol problems. Furthermore, the findings suggest that parenting style is important for emotional regulation. A connection was found between controlling parents and poor emotion regulation in the children (Haverfield & Theiss, 2017). Children who grow up in very authoritarian homes have difficulty regulating emotions and behaviour. Controlling parents will to a greater extent tell what the child should do and feel, rather than discovering what the child is feeling (Haverfield & Theiss, 2017).

Haverfield & Theiss (2014, 2020) provide support for the importance of communication as a risk factor. They found (2020) that the dimensions of parental communication uniquely associated with the potential markers of adolescent resilience. Adolescents from families with parental alcohol misuse behaved more impulsively when parents’ attempted emotion coaching. However, they had better emotion regulation in response to emotion dismissing communications compared to their peers from sober families.

### Alcohol Misuse as an Influence on Family Dynamics

Four of the included studies focused on alcohol misuse as an influence on family dynamics (Coyle et al., 2009; Lee & Williams, 2013; Park & Schepp, 2018; Hall, 2008). Coyle et al. (2009) examined patterns in family functioning that could potentially act as protective factors for families with alcohol misuse. The study focused on parenting style, current alcohol misuse, supportive relationships outside the home, early stressful situations and race. Findings showed that the families who had above average functioning also had above-average upbringing in terms of parent-child involvement and discipline. Families with average or below average functioning also had below average upbringing. The results suggest that families with parents who misuse alcohol may be differentiated by family function. Although no association between demographic characteristics and family function was found, they found that race had a significant impact. African American families were more likely to be in medium to high functioning, compared to “white” and Native American families. Coyle et al. (2009) believe that this may be due to religion and belief as African American families reported to a greater extent that believing in a god and having faith helped them in difficult times.

Lee & Williams (2013) investigated the association between lack of belonging and depression in adult children of alcohol-misusing parents. The findings showed that lack of belonging is the biggest risk of developing depression in these adults. Parental mental health in addition to alcohol misuse also had an impact on adult child depression. Lee & Williams (2013) believe that a stronger sense of belonging can help these adults recognise positive aspects of their personal relationships and thereby reduce stress and counteract depression. One factor that can affect the sense of belonging is childhood living conditions. Park & ​​Schepp (2018) and Hall (2008) found that living with other family members who are healthy was a protective factor. When it was chaotic at home, it was important to be able to trust other family members such as a sober parent or sibling. Relationship, both with the sober parent and the parent with alcohol use disorder, emerged as an important factor. Most adult children of alcohol-misusing parents indicated they valued the love and encouragement they received from their sober parent throughout their upbringing and credited this as vital for their positive development. Violence is considered a serious risk factor, but while Lee & Williams (2013) found that parents’ alcohol misuse was moderately associated with episodes of domestic violence, depression in adult children of these families was only minimally associated with domestic violence.

### The Importance of Social Support

Nine of the included studies examined the importance of social support (Moe et al.,^2007^; Hall, 2008; Park & Schepp, 2018; Park & Schepp, 2017; Bugeja & Galea, 2022; Tamutiene & Jogaite, 2019; Hebbani et al., 2020; Haverfield & Theiss 2014; Haverfield & Thiess, 2016). Moe et al. (2007) found that participants felt having someone they trust and with whom they express their feelings was a protective factor. A number of participants in a study by Park & ​​Schepp (2018) also credited social support from a trusted individual with helping reduce stress in everyday life and contributing to a sense of security. Participants in a study by Bugeja and Galea (2022) described that the difficult relationship with their alcohol-misusing parent drove them to build stronger relationships with siblings. It was common for them to seek comfort and support from each other. Additionally, Tamutiene and Jogaite (2019) found that children who were able to share their problems with siblings, grandparents or aunts felt better. Growing up with a parent with alcohol use disorder also led them mature early and appreciate support from others, as their social support was mainly provided by friends.

Hebbani et al. (2020) found that availability and accessibility to family and community support and religious rituals were associated with greater resilience among young adults of alcohol-misusing parents. Those who had high scores on resilience and higher self-acceptance had good family and community support and took an active part in family and community sanctioned religious activities.

Haverfield & Theiss (2014) also found that social support was a protective factor. Participants in the study reported that sharing their story with others who can relate to it can help reduce loneliness. Internet-based support groups can help deal with stigma in this manner.

### Interventions

Two studies were identified that deal with interventions for children of alcohol-misusing parents (Hyun et al., 2010; Grance-Cleveland & Mays, 2008).

### The Importance of Behavioural Therapy

The first study aimed to investigate whether a 10-week behavioural intervention could improve self-concept, resilience and depression in children growing up with parental alcohol misuse (Hyun et al., 2010). Participants were tested in self-concept, resilience and depression, before and after the behaviour therapy intervention. The findings showed no change in depression or self-concept in the experimental group post intervention, but resilience in this group had increased significantly. In the control group, there was no change in any of the outcomes. According to Hyun et al. (2010), the increase in resilience among the individuals in the experimental group indicated the participants were using more effective coping strategies after the intervention than before. These positive results show that behavioural therapy can help identify participant strength and potential.

### The Importance of School-Based Support Groups

The second intervention study (Grance-Cleveland & Mays et al., 2008) focused on school-based support groups for adolescents who had substance using family members. After the intervention, which lasted for 14 weeks, knowledge about drug use increased considerably in the experimental group. There was no difference in mental symptoms for girls between the experimental group and the control group, but for boys, there was a 15% symptom reduction in the experimental group after the intervention. For both sexes, the findings showed a 20% increase in positive mood after the intervention. Additionally, girls showed a significant increase in coping strategies compared to boys after the intervention. Participants in the experimental group reported after the intervention that they drank less alcohol compared with the control group, which had up to an 88% increase in wine consumption and up to 32% consumptions of other alcohol.

## Discussion

This study analysed relevant evidence regarding resilience, risk and protective factors, and coping among children of alcohol-misusing parents. The results revealed themes of biological and genetic differences, children’s innate factors, attachment and communication, family dynamics and social support. The main findings identified risk and protective factors that can impact resilience in children raised in families with alcohol misuse. Children of alcohol-misusing parents have more risk factors such as emotional problems, lower academic performance, lower self-esteem and more behavioural difficulties compared to other children. The most important protective factor highlighted in our study is social support. Communicating with friends, grandparents or having a sober parent or sibling to talk to and trust are all examples of this protective factor. Further, children’s inherent characteristics, engaging in sports and maintaining a healthy lifestyle, and having faith were also found to be protective factors. Children of alcohol-misusing parents are more likely to develop depression, alcoholism and low self-esteem. Resilience cannot be understood without considering factors at all levels. Children of alcohol-misusing parents are a vulnerable group that needs further attention. Research focusing on children of parents who misuse alcohol is limited and there are few intervention studies on this topic.

The evidence analysed demonstrated that factors on genetic, family and social levels impact children of alcohol-misusing parents and increase the chances that they may develop alcohol problems themselves. Genetic differences were found in children from families with alcohol misuse compared to children from sober families which may indicate that resilience has a genetic transmission, although this is not a wholly reliable finding (Ossola et al., 2020). At the genetic level, children growing up with parental alcohol misuse have higher levels of dopamine D2 (DRD2) which is associated with being more prone to developing alcohol misuse. Differences in genes and brain function can be seen as risk factors based on our findings. These finding support a study by Edenberg & Foroud (2013) illustrating that alcoholism is a complex genetic disease with variations in many genes affecting risk.

Emotion regulation theory and attachment theory are particularly relevant frameworks for discussing the findings and emotional outcomes for adult children of alcohol-misusing parents (Pascuzzo et al., 2015). Considering attachment from a regulatory perspective, these genetic differences can be developed based on personal characteristics. Based on emotional regulation theory and attachment theory, resilient personal characteristics would not develop if there is no secure connection and regulatory support present at an early age (Brandtzæg et al., 2014). Secure attachment supports mental health, the child has access to their feelings and can seek closeness and comfort when the needed. Children growing up with parental alcoholism may lack opportunity to seek comfort from their parents when needed, this can negatively affect their attachment style. In attachment theory, the management of emotions plays an important role. However, emphasis on the importance of meeting the child’s need to be emotionally regulated does not mean the goal is for the child to always seek help from others. The aim is for the child to eventually achieve a balance between self-regulation and regulation with others, these are prerequisites for being able to function well emotionally and socially later in life (Brandtzæg et al., 2014). Taking into consideration the findings described above, children who grow up with parental alcoholism will likely struggle emotionally, socially and with self-regulation when they become adults.

Protective factors identified at the individual level included belief in oneself, self-confidence and positive coping strategies. Individual qualities can also be seen in the context of social support, for example, those who understand when they need emotional support implement measures to obtain it. Alternately, temperament, impulsivity and attention were traits identified as associating with the development of alcoholism and mental health problems. Furthermore, children of families who misuse alcohol use less effective coping strategies compared to other children from families without alcohol misuse. Gender differences were identified at both individual and social levels. Gender differences were notable in intervention studies in emotional and behavioural problems as well as in coping and resilience. These findings suggest that boys are more vulnerable to developing behavioural difficulties and alcohol use compared to girls. This is in line with research showing that women are more likely to develop depression and eating disorders than men, while men were more likely to develop antisocial personality disorders and alcohol use (Rodney et al., 2000).

One factor related to children of parents with alcohol misuse potentially developing an alcohol use problem themselves has not been highlighted much in the empirical literature. Alcohol and substance use in adolescents is often associated with triggers such as mental health problems, disinhibition, impulsivity, and sensation-seeking (Rodney et al., 2000; Spear, 2000). Ensminger et al. (1982) found that men who are shy at an early age and later show extravagant aggressive behaviour are more likely to develop heavy drug use. But for women it seems that challenging family relationships are a risk factor. These findings suggest that there may be different developmental pathways that lead to drug use. Flannery et al. (1994) on the other hand, found gender differences when it came to developing drug use. Morgan et al. (2010) studied how gender differences lead to offspring psychiatric illness in children of alcohol-misusing parents. The results show significant differences in the types of mental health issues in males versus females, and generally female children of woman with alcohol use disorder apper to be at higher risk of mental health problems. Because the research on gender differences is currently limited, as of yet it is unclear what role it has for prevention in children of alcohol-misusing parents. More focus must be placed on investigating the role of gender differences in order to develop tailored prophylactic strategies to prevent children of alcohol-misusing parents developing alcohol-misuse themselves.

At the parent and family level, Haverfield & Theiss (2017)’s findings indicated that attachment and communication are important factors for healthy child development. This is in line with the theories that have been elucidated throughout the discussion, where regulatory support and early affiliation are paramount in the development of “internal working models” (frameworks shaping perceptions of oneself and others that guide relationship behaviors and expectations) (Bowlby, 1982; Bretherton, 1991). Internal working models” may be important for the development of resilience given the connection between “internal working models” and emotional resilience. Lack of awareness of the factors that increase the risk of negative outcomes among children of alcohol-misusing parents is problematic (Leonard & Eiden, 2007). According to our findings, children from families that have poor connections, poor interaction and lack of communication will fare worse. Parents with alcohol misuse have been shown to be less emotionally present for their children (Osofsky & Thompson, 2000). Despite this, children from large families in collectivist cultures may thrive in the context of having other family members to rely on.

Social support is a clear protective factor as evidenced in both the qualitative and quantitative studies, but how we can we ensure children of alcohol-misusing parents get the social support they need? Children of alcohol-misusing parents often are subject to social stigma and may therefore withhold information about their family environment. This can make it difficult to identify and assist these children. The challenge is to discover them as early as possible. Therefore, the school plays an important role. Schools often have a low tolerance for acting out and problem behaviour in children, this can lead to punitive processes being initiated that can harm the child instead of helping. At the same time, it can be difficult to distinguish if the child’s behaviours are related to a problematic home environment or intrinsic traits (Befring & Duesund, 2012).

Interventional findings show that behavioural therapy and school-based support groups have a positive effect on children and adolescents of alcohol-misusing parents (Hyun et al., 2010 and Grace-Cleveland & Mays, 2008). Based these findings, there is a need to strengthen supportive programmes for children of alcohol-misusing parents. Most of the intervention studies we reviewed focused on working with characteristics of the child, but it may be just as important to emphasize building relationships with other healthy family members due to social support’s protective role. Despite societal investments in intervention work, many growing up in families with alcohol misuse will experience neglect. The effects of this are not only emotional problems, but also violence and poverty. These are dire consequences, not only for the children themselves but also society at large. Such consequences necessitate identifying affected children and intervening in order to mitigate harm. In this regard, low-threshold services and universal follow-up in kindergartens and schools can act as a key safety net (Nordanger & Braarud, 2017). Severe neglect’s visible signs can make it straightforward to detect, but there are many families who seem functional despite neglect being present. Therefore, it is vital that public services have sufficient competence and confidence to ask and ascertain if neglect is an issue.

### Strengths, Limitations and Future Directions

This scoping literature review aimed to provide an overview of research regarding children in families with alcohol-misuse. The purpose of a scoping review is to summarise available empirical research on a topic in order to provide an overview to contribute to new knowledge. This is considered to be one of the main strengths of a literature study (Aveyard, 2014). Although we conducted a systematic literature search, it is possible that we have not uncovered all the relevant literature, as there are a number of databases that could have been used to identify relevant articles. We do not know if including additional databases or choosing alternate databases would have yielded further relevant literature. This is the main limitation of the study. However, the selected databases are well established for medical, psychological and social research, and our search encompassed 14 years of empirical research.

Literature reviews aim to identify research gaps in the empirical literature. We found only 10 qualitative studies dealing with children and adolescents in families with alcohol misuse. As qualitative studies are seen as an important for understanding the meaning behind a phenomenon, it is surprising how few qualitative studies were found. Qualitative studies also help to give a voice to a phenomenon. Qualitative studies in children of parents who misuse alcohol would therefore be in line with the child’s right to participation and the principle of “the child as an actor” (UN General Assembly, 1989). It is the children’s opinions and experiences that can inform us about the experience of growing up in alcohol-misusing families and what interventions are helpful. In addition, a predominance of included studies was at a biological or individual level rather than a parent or family level.

More research is needed on family dynamics in order to gain a greater understanding of this group. The family has a large impact on the child and there is a need for more qualitative studies with this as a focus. Additionally, both biological and environmental factors can lead to poor upbringing or care and therefore biological and environmental research is also necessary. Furthermore, we found few intervention studies among children of parents with alcohol misuse, more interventional research is necessary. Our findings show that there is an indication that boys and girls respond differently to intervention and treatment. Some gender differences were identified, but despite this, many studies did not take gender differences into account. There is a need for further research focused on how gender may affect outcomes in this group, this would contribute to a more complete understanding of resilience and the impact of interventions in children of parents with alcohol misuse. Considering the social level, few social factors were identified. For example, none of the studies addressed the importance of the school and child welfare when it comes to public support. There is need for more research when it comes to what the school and child welfare programmes can do to assist this group in addition to research on what interventions increase knowledge on child welfare.

### Implication for Practice

Our findings from this scoping review contribute to the knowledge base on resilience among children of parents with alcohol misuse. The findings shed light on this important topic and on the status quo among this vulnerable group.

Alcohol misuse is highly stigmatized. Increased knowledge in research, clinical practice and society at large can reduce stigma for affected families and contribute to open communication on this topic. Our findings may be used as a springboard for children of parents with alcohol misuse and their families to express their feelings. Experts within the public sphere such as teachers, childcare workers and other practitioners can use our findings to tailor their practices for educating affected children and families and helping them develop new coping mechanisms and social supports.

Intervention programs can go a long way towards buffering negative experiences, offering healthier alternative ways of living and coping. Our analysis illustrates the importance of social support as a protective factor for children of alcohol-misusing parents. Thus, intervention centered on educating families about the essential nature of social support can also improve quality of life and help safeguard against future alcohol use disorder.

## Supplementary Information

Below is the link to the electronic supplementary material.

ESM 1 (DOCX 16.1 KB)

## Data Availability

Not Applicable.

## Appendix

**Table 1:** Characteristics of included studies

Author and year	Sample size	Research design	Country	Aim	Key findings
Bugeja and Galea (2022)	6 Adult children of alcohol-misusing parents	Qualitative	Malta	To explore the lived experiences of Maltese adult children of alcohol-misusing parents.	A number of findings resulting from this study illustrate resilience and its associated skills, as well as who moves on to lead successful lives, despite their past hard life.
Coyle et al. (2009)	674 Families	Quantitative	Canada	Family functioning that may protect families from the negative impact of alcohol misuse.	The study reveals a continuum of family functioning associated with parenting, as well as children`s perception of teachers caring, and race.
Drapkin et al. (2015)	465 Students	Quantitative	United states	Exploring if coping styles predict alcohol use, positive or negative life events, and depression in adult children of alcohol-misusing parents.	They had more alcohol problems, more negative life events and less effective coping styles compared with other students. No different in depression.
Einsberg et al. (2010)	467 Children	Quantitative	United States	Relationships between externalising problems and personality ego resilience.	Relationships between temperament and externalising problems were stronger in and sometimes only found in children growing up in alcohol misuse, especially males.
Gance-Cleveland & Mays (2008)	109 Students	Quantitative	United States	The purpose of this study was to evaluate school-based support groups for adolescents with substance-misusing parents.	Significant improvements in knowledge of substance misuse were noted. Gender differences in coping and health outcomes. The participants reported less alcohol use than control.
Hall (2008)	32 Students	Qualitative	United States	Examine how kin and fictive kinship relationships influence adult children`s upbringing.	Results from in depth interview reveals the importance of social support from fictive family to help them cope.
Haverfield & Theiss (2020)	30 Families	Qualitative	United States	This study examines how features of parental communication may encourage or undermine the development of resilience in adolescents from alcoholic families.	The findings show that adolescents from families of parental alcohol misuse is more impulsive under parent’s emotion coaching and emotion dismissing communication and better at emotional regulation in response to emotion dismissing communications.
Haverfield and Theiss (2017)	60 Families	Qualitative	United States	Examines how communications between parents and children can impact resilience in children of alcohol-misusing parents compared to other children.	Parental responsiveness was positively associated with adolescent emotion regulation in both interactions, and parental control was positively associated with adolescent impulsivity in the negative interactions. For families with alcohol misuse, parental control was positively associated with adolescent impulsivity in the positive interactions.
Haverfield and Thiess (2016)	622 Adult children of alcohol-misusing parents	Quantitative	United States	Examined the severity of a parent`s alcoholism and family topic avoidance about alcohol as two factors that are associated with family stigma.	The severity of the parents’ alcohol misuse and communication failure in the family was associated with stigma in women. For men, the main factor linked to stigma was just the family`s failure to communicate about alcohol-related issues. Those who feel stigmatised have an increased degree of depressive development and lower self-esteem and resilience.
Haverfield & Theiss (2014)	811 Thematic devices	Qualitative	Unites States	This study addresses what adults share about their experiences of growing up in alcoholic families on online support groups.	Social support was the topic that was most discussed. Another topic was how growing up still affects them in adulthood and how they struggle to cope with everyday life. Low self-esteem was mentioned and a feeling of anger towards their alcoholic parents.
Hebbani et al. (2020)	331 children of alcoholics	Quantitative	India	This study aims at understanding the relationship between resilience and positive psychological factors among healthy young adult of alcohol-misusing parents.	Children who obtained high scores on measures of resilience had a good family and community support. Those who took an active part in family and community sanctioned traditional religious activities and rituals had higher self-acceptance. Also focused on positive coping strategies.
Heitzeg et al. (2008)	28 Children	Quantitative	Unites States	Identify neural activation mechanism that may mark protection or vulnerability to alcohol misuse in children.	Children of alcohol-misusing parents are more vulnerable to a serious alcohol use than other children. The resilient group showed an active emotional motor function, in response to emotional stimuli which may be a protective factor in this group. The vulnerable group displayed a pattern consistent with active suppression of affective responses.
Hyun et al. (2010)	317 Young men	Quantitative	South Korea	Effects of cognitive behavioural therapy aimed at enhancing the resilience of high-risk adolescents with alcohol-dependent parents.	Significantly higher resilience (coping) in the group with alcohol-misusing parents. Results indicate that the developed CBT program might be effective for improving resilience of adolescent with alcohol-misusing parents.
Klostermann et al. (2011)	619 Students	Quantitative	United States	Examined whether adult children of alcohol-misusing parents report different coping behaviours compared with other adults, and if these coping behaviours were related to depressive mood symptoms in adults with familial alcoholism.	Adults from familial alcoholism reported more depressive symptoms and less effective coping behaviours compared with other adults. Adults from parents with alcohol use disorder also had more drug related problems.
Lee & Cranford (2008)	482 Adolescent	Quantitative	United States	Examines the influence of parental alcohol misuse and resilience on externalising problems in young adults of parents with an alcohol use disorder.	Parental problem drinking was directly associated with externalising and internalising behaviours.
Lee & Williams (2013)	206 Adult children of alcohol-misusing parents	Quantitative	United States	Relationships between parental alcoholism, sense of belonging, resilience, and depressive symptoms among Koreans in the U.S.	Sense of belonging were the most powerful, and resilience the second most important factor for resisting depressive symptoms associated with parental alcoholism.
Moe & Wade (2007)	50 Children	Qualitative	United States	The goal is to use the “Betty Ford Children’s Program” to get children to describe what it means to be resilient and what it takes to have a good life despite growing up in alcoholic homes.	Three important concepts were identified to create a good life. Knowledge, expression of emotions and making the right life choices.
Mushonga & Van Breda (2021)	15 Students	Qualitative	South Africa	The aim is to is to explore the interactions of resilient adult children of alcohol-misusing caregivers with nonhuman systems to understand what nonhuman systems they engage with and how these interactions operate to promote resilient outcomes.	Participants report extracting empowering messages from these systems and engaging with the message and/or system in ways that build their capacity to withstand or overcome the negative impact of growing up with an alcohol- using caregiver.
Namkoong & Cheon (2008)	45 Adolescent	Quantitative	South Korea	The aim is to find out if children of alcohol misusers have genes that give a higher risk of developing alcohol problems themselves.	The findings show that children of alcohol-misusing parents have a larger proportion of genes related to alcohol dependence.
Park & Schepp (2018)	22 Adult children of alcohol-misusing parents	Qualitative	South Korea	This study examines what adult children of alcohol-misusing parents think is an influencing factor for resilience.	Identified four levels impacting resilience in adult children of alcohol-misusing parents. Important protective factors identified included social support, economy, self-esteem and knowledge.
Park & Schepp (2017)	20 Adult children of alcohol-misusing parents	Qualitative	United States	To study Korean adults of alcohol-misusing parents’ psychosocial adaptation throughout adolescence.	Those interviewed showed the same patterns in how they had adapted throughout their upbringing. Six levels of adaptation were identified.
Smith et al. (2006)	180 Families, 293 Children	Quantitative	United States	Relation between parent’s alcohol misuse and the children’s coping strategies and coping efficacy.	Some of the coping strategies were moderated by parent’s alcohol misuse.
Spivakovskaya & Lutsenko (2021)	58 adult children of misusing parents and 50 other adults	Combined quantitative and qualitative	Russia	The purpose of this study was to create a model of resource factors which would allow people with alcohol-addicted parents to overcome the negative emotions they experienced.	The results show that a taboo on expressing emotions, and demonstrating external well-being were associated with being unable to recognise current negative emotions and with avoiding problems. Resource factors which allowed these subjects to overcome their negative emotions included: communication with relatives and friends; keeping a diary of emotions; and participating in recovery programs.
Tamutiene & Jogaite (2019)	23 Children	Qualitative	Lithuania	The aim of this study was to learn to whom children disclose experience of harm caused by their parents with alcohol problems.	The disclosure of problems was significant for the children who subsequently perceived a sense of community. The sharing of their experiences made it easy for the children to overcome their struggles.
Wong et al. (2018)	715 Children	Quantitative	United States	Examined the relationship between sleep rhythmicity, behavioural control and resilience in children of alcoholics.	Good sleep and higher self-regulation act as resource factors for young adults, regardless of parental alcoholism status.
