# Supplementary material for: Resilience, Risk and Protective Factors in Children and Adolescents Growing Up with Parental Alcohol Misuse
Source: J Child Adolesc Trauma. 2025 Oct 27;19(2):385–400. doi: 10.1007/s40653-025-00776-0 (PMC13190856; doi:10.1007/s40653-025-00776-0)
Supplement: Supplementary file 1 — (DOCX 16.1 KB) [file 40653_2025_776_MOESM1_ESM.docx]

12.09.25

Dear Editor,

Thank you very much for considering the acceptance of our revised manuscript entitled “Resilience, Risk, and Protective Factors in Children and Adolescents Growing Up with Parental Alcohol Misuse” for publication as a scoping review in the Journal of Child and Adolescent Trauma. We are also very grateful for the reviewers’ minor corrections to our manuscript. We believe their insightful comments have helped strengthen the overall quality of our paper.

Our manuscript totals 39 pages, including figures, boxes, and references. In order to comply with the journal’s 40-page limit, we would like to move “Table 1: Characteristics of Included Studies” to the appendix. The authors have carefully addressed the reviewers’ suggestion to revise the language of the manuscript, and the text has been thoroughly edited to improve clarity and readability.

We confirm that this manuscript has not been published elsewhere and is not under consideration by any other journal. Furthermore, none of the authors have any conflicts of interest to disclose in relation to this study.

Thank you very much for your time and consideration.

Best regards,

Kristel Svendal

Master in Child Protection, University of Bergen, Norway
